# MacCallum v. Burdett and Others

**Published:** 1920-11-27

**Authors:** 


### MacCallum v. Burdett and Others.

This case was taken before the Lord Chief Justice and a special jury on Tuesday, November 23. Before its opening, counsel for the defendants announced that in view of the death of Sir Henry Burdett, the principal defendant, last April, and the impossibility now of taking instructions from him, the other defendants desired to withdraw the pleas of justification and fair comment. Subsequently it was announced that agreement had been reached, and that the defendants would pay the plaintiff £500 and give her an indemnity for costs and expenses, all charges made against her being unreservedly withdrawn.

Miss Margaret Isabelle Holden has been appointed superintendent of the Children's Home under the Devonport Board of Guardians. She has had a similar post under the Lancaster Guardians.

